# Hubness of strategic planning and sociality influences depressive mood and anxiety in College Population

**DOI:** 10.1038/s41598-017-18189-x

**Published:** 2017-12-19

**Authors:** Je-Yeon Yun, Yoobin Choi, Yoonhee Kwon, Hwa Young Lee, Soo-Hee Choi, Joon Hwan Jang

**Affiliations:** 1 Seoul National University Hospital, Seoul, Republic of Korea; 2 Yeongeon Student Support Center, Seoul National University College of Medicine, Seoul, Republic of Korea; 3 Department of Psychiatry, Seoul National University Hospital, Seoul, Republic of Korea; 4 Department of Psychiatry, Seoul National University Health Service Center, Seoul, Republic of Korea; 5 Department of Medicine, Seoul National University College of Medicine, Seoul, Republic of Korea

## Abstract

Depressive mood and anxiety can reduce cognitive performance. Conversely, the presence of a biased cognitive tendency may serve as a trigger for depressive mood-anxiety. Previous studies have largely focused on group-wise correlations between clinical-neurocognitive variables. Using network analyses for intra-individual covariance, we sought to decipher the most influential clinical-neurocognitive hub in the differential severity of depressive-anxiety symptoms in a college population. Ninety college students were evaluated for depressive-anxiety symptoms, Minnesota multiphasic personality inventory-2(MMPI-2), and neuro-cognition. Weighted and undirected version of the intra-individual covariance networks, comprised of 18 clinical-neurocognitive variables satisfied small-worldness and modular organization in the sparsity range of *K* = 0.20–0.21. Furthermore, betweenness centrality of perseverative error for the Wisconsin card sorting test was reduced in more depressive individuals; higher anxiety was related to the increased betweenness centrality of MMPI-2 clinical scale 0(Si). Elevated edge-betweenness centrality of covariance between the MMPI-2 clinical scale 7(Pt) versus commission error of the continuous performance test predicted more anxiety higher than depressive mood. With intra-individual covariance network of clinical-neurocognitive variables, this study demonstrated critical drivers of depressive mood[attenuated influence of strategic planning] or anxiety[domination of social introversion/extroversion, in addition to the influence of compulsivity-impulsivity covariance as a shortcut component among various clinical-neurocognitive features].

## Introduction

About 17–31% of the college students who are not receiving psychiatric services have reported significant depression experience at some point^1,2^. In the case of anxiety symptoms, the prevalence rate is higher, and about 10% of university and graduate students have reported significant anxiety symptoms at some time during their school years^2^. Experience with depression and anxiety can temporarily reduce an individual’s concentration, memory, and performance of high-level cognitive functions, which can lead to a decrease in academic performance and secondary life stress^3^. Moreover, when there is a cognitive distortion of high-level executive functions such as reasoning and judgment, individuals become more susceptible to stress and more easily exposed to experiences of depression and anxiety^1,2,4^. However, most clinical studies have only explored group-wise correlations between depressive mood, anxiety, and experienced cognitive dysfunction, or have calculated quantitative differences in cognitive functioning ability between patients diagnosed with psychiatric disorder versus normal controls^5–7^.

Therefore, to acquire sufficient information on the dynamics between various aspects of diverse psychopathology and cognitive functioning, inter-individual network-based approaches such as Gaussian graphical model^8^ (partial correlation network in which edges represent conditional independence relationships among the nodes; for ordinal or continuous variables)^9^ regularized using graphical Lasso^10^, and Ising model (assuming that activation of a node is dependent on the activation of its neighbouring nodes; for dichotomous variables)^11^ fitted using eLasso have been developed. These network estimation methods are readily implemented in the R package qgraph^12^ and IsingFit (https://cran.r-project.org/web/packages/IsingFit/index.html). Having been combined with graph theory metrics^13,14^, hierarchical and segregated relationships among emotional-behavioural problems^15^, psychiatric symptoms^16^, psychiatric diagnoses^17^ have been reported. Trans-diagnostic^9,11^ deciphering of symptom-to-symptom interaction as well as disorder-specific^18–21^ identification of key drivers in the symptom network have been also conducted. Furthermore, for depression and anxiety disorder, key features of diagnosis specificity^22^, stressor-symptom interaction^23^, clinical course^24^, in addition to the interplay among clinical symptoms and neurocognitive variables^25,26^ have been uncovered. However, to use the results of these network approach for personalized medicine and treatment, construction of intra-individual network is required^11^.

Then, how then can researchers identify the dynamics of depressive mood, anxiety, personality traits, and the subdomains of neurocognitive performance at the individual level, rather than at the group level, and recognize the key drivers of such mental states? The notion of intra-individual variability originally described inconsistencies of neurocognitive performance within a person either (a) in the same task across multiple assessments over a long period of time, (b) over multiple trials of a single task for one occasion or over short periods of time, or (c) for diverse neuropsychological tasks on a single session. Using the observational frame of (c), in this study, we sought to explore more sensitively the changed patterns of dynamics among nonverbal neurocognitive capacity, including attention, processing speed, working memory, and executive functioning [set shifting and cognitive flexibility]^27,28^. Taking one step further, in the context of a network perspective of personality organization^29^, we explored the inter-connectedness of cognitive, affective and behavioural components of each participant, measured using nonverbal neurocognitive tasks, self-reported depressive mood and anxiety, and the Minnesota Multiphasic Personality Inventory-2 (MMPI-2). Moreover, to explore the dynamics of cognitive, affective, and behavioural components at the individual level, each set of inter-variable covariance was not intermingled into a summary score of ‘intra-individual variability’ but preserved intact to construct an intra-individual covariance network^30,31^. Using a graph theory approach^32^ for the intra-individual covariance network, we aimed to decipher the most influential clinical-neurocognitive hub in the differential severity of depressive-anxiety symptoms in a college population.

## Results

### Sample characteristics

The mean age of the sample was 24.1 (SD = 4.3) years, and 63.3% of participants were women. All participants were undergraduate or graduate students at the time of study participation, and had received an education longer than 12 years. None of the subjects were taking nay psychiatric prescription drugs at the time of study entry. Furthermore, the means and standard deviations of the 18 measures included in this study are described in Table 1.

**Table 1:** Mean and standard deviations of clinical, and neuropsychological measures.

Variable (*n* = 90)	M	SD
Beck depression inventory	24.6	10.8
Beck Anxiety Inventory	16.8	11.1
Trail making test part A: reaction time	21.2	5.7
Trail making test part B: reaction time	48.6	13.1
Continuous performance test: omission error	1.7	4.6
Continuous performance test: commission error	14.3	7.5
Digit span:forward	11.7	2.2
Digit span: backward	9.6	3.1
Wisconsin card sorting test: perseverative error	6.6	5.3
T-score of MMPI-2 clinical scales: 1(Hs)	55.8	11.4
T-score of MMPI-2 clinical scales: 2(D)	66.4	11.8
T-score of MMPI-2 clinical scales: 3(Hy)	57.6	10.5
T-score of MMPI-2 clinical scales: 4(Pd)	56.2	11.7
T-score of MMPI-2 clinical scales: 6(Pa)	55.6	10.9
T-score of MMPI-2 clinical scales: 7(Pt)	63.4	12.6
T-score of MMPI-2 clinical scales: 8(Sc)	58.3	11.2
T-score of MMPI-2 clinical scales: 9(Ma)	46.9	9
T-score of MMPI-2 clinical scales: 0(Si)	62.5	12.6

### Global network characteristics

**Figure 1:**
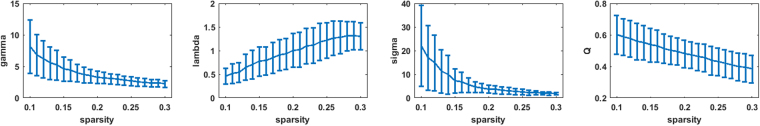
Global network characteristics of the intra-individual variability-based network at sparsity range of K = 0.10–0.30: (**A**) gamma (normalized clustering coefficient), (**B**) lambda (normalized characteristic path length), (**C**) sigma (small-worldness), and (**D**) Q (modularity).

In the optimal sparsity range of K = 0.20–0.21, for more than 95% of the participants, the weighted and undirected intra-individual covariance networks consisting of 18 clinical-neurocognitive variables satisfied the (1) small-world organization (*sigm*a > 1), (2) modular organization (Q > 0.3; *K* = 0.10–0.21), and showed (3) network connectedness [more than 80% (>15) of nodes from a total of 18 nodes were connected to at least one other nodes; *K* = 0.20–0.21]. Group-averaged mean and standard deviation for a total of four global network characteristics including normalized clustering coefficient (*gamma*), normalized characteristic path length (*lambda*), small-worldness (*sigma*) and modularity (*Q*) values across the searched sparsity range of *K* = 0.10–0.30 are illustrated in Fig. 1.

### Betweenness centrality vs. severity of depressive mood or anxiety

**Figure 2:**
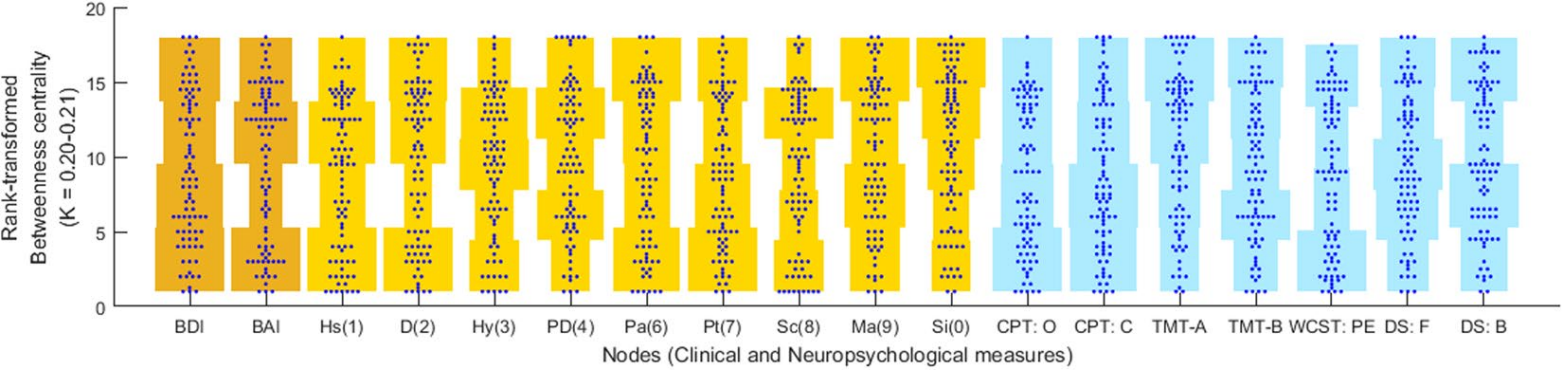
Distribution of the rank-transformed betweenness centrality values for a total of 18 nodes in the weighted, undirected version of the intra-individual variability-based network at sparsity range of K = 0.20–0.21, within which three conditions of (1) >15 nodes (from a total of 18 nodes) are connected, (2) modularity (Q) >0.3, and (3) small-worldness (sigma) >1 were satisfied in >95% of the participants (*n* = 90). Illustration was conducted using distributionPlot.m running in the Matlab software. Abbreviations: BAI, Beck Anxiety Inventory total score; BDI, Beck Depression Inventory total score; 1(Hs), MMPI-2 clinical scale 1(Hypochondriasis); 2(D), MMPI-2 clinical scale 2(Depression); 3(Hy), MMPI-2 clinical scale 3(Hysteria); 4(Pa), MMPI-2 clinical scale 4(Psychopathic deviate); 6(Pa), MMPI-2 clinical scale 6(Paranoia); 7(Pt), MMPI-2 clinical scale 7(Psychasthenia); 8(Sc), MMPI-2 clinical scale 8(Schizophrenia); 9(Ma), MMPI-2 clinical scale 9(Hypomania); 0(Si), MMPI-2 clinical scale 0(Social introversion); CPT-O, omission error of the continuous performance test; CPT-C, commission error of the continuous performance test; TMT-A, reaction time of the trail making test part A; TMT-B, reaction time of the trail making test part B; WCST-PE, perseverative error of the Wisconsin card sorting test; DS-F, forward span of the digit span test; DS-B, backward span of the digit span test.

The distribution of the rank-transformed betweenness centrality values per participant averaged in the *K* = 0.20–0.21 is shown in Fig. 2, drawn using distributionPlot.m (https://kr.mathworks.com/ matlabcentral/fileexchange/23661-violin-plots-for-plotting-multiple-distributions–distributionplot-m-) within Matlab R2014b software. When the rank-transformed betweenness centrality value is low, the original betweenness centrality value of a given node [clinical-neurocognitive measure] would be higher than other nodes in the intra-individual covariance network of that participant.

**Figure 3:**
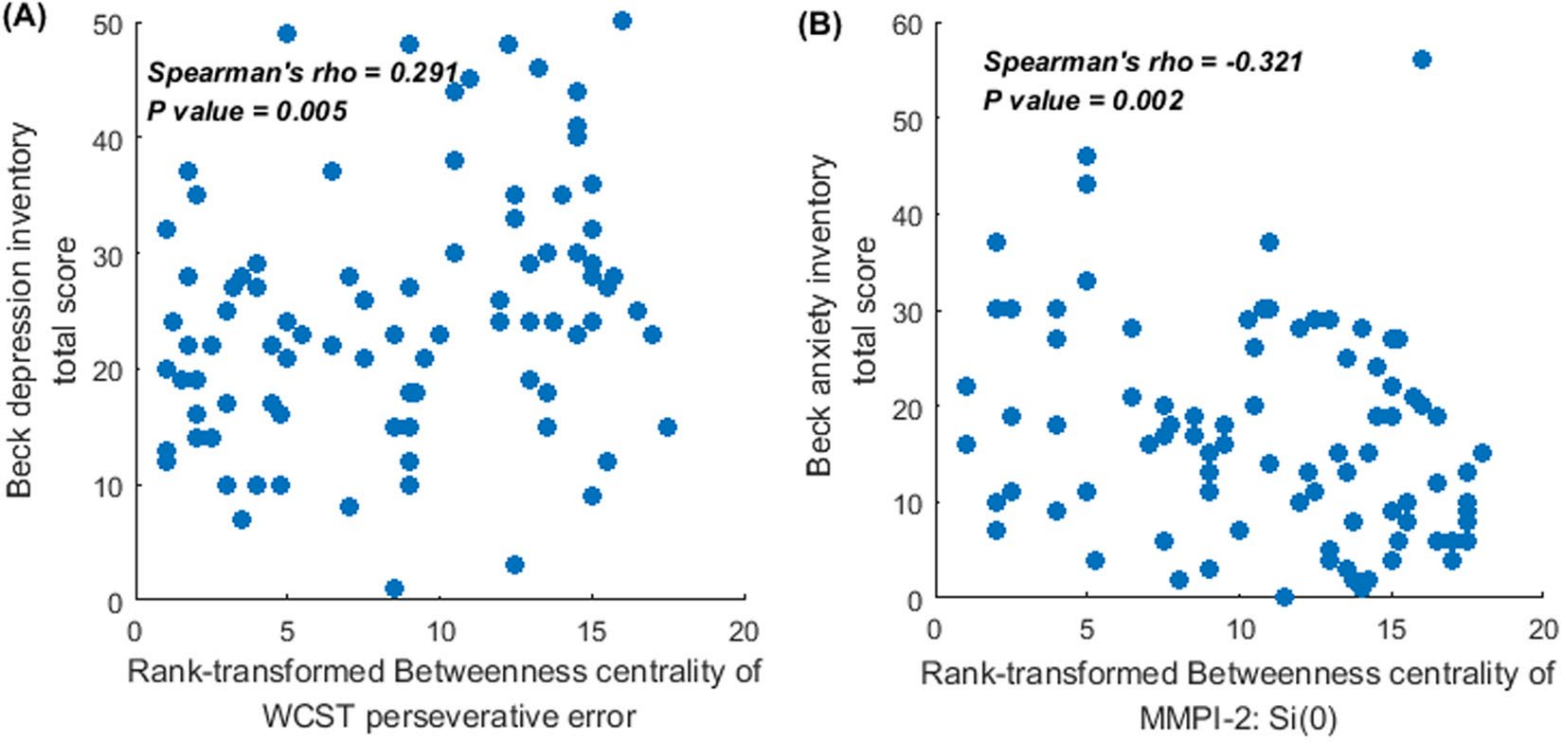
Relationship between the rank-transformed betweenness centrality [calculated from the intra-individual variability network (undirected and weighted version in the sparsity range of K = 0.20–0.21)] of (**A**) Wisconsin card sorting test: perseverative error with severity of depressive symptom, and (**B**) T-score of Minnesota multiphasic personality inventory clinical scale: Si(10) with severity of anxiety symptom (*p* < 0.01)

Among these nodes, rank-transformed betweenness centrality of Wisconsin Card Sorting Test (WCST) perseverative error demonstrated statistically significant correlation with the total score of Beck Depression Inventory (BDI; *Spearman’s rho* = 0.291, *p* = 0.005; Fig. 3(A)). Rank-transformed betweenness centrality of MMPI-2 clinical scale Si(0) demonstrated a statistically significant relationship with total score of Beck Anxiety Inventory (BAI; S*pearman’s rho* = *−*0.321, *p* = 0.002; Fig. 3(B)). Otherwise, no statistically significant correlation was found (all *p > *0.01).

### Edge betweenness centrality vs. depressive mood-anxiety dynamic

**Figure 4:**
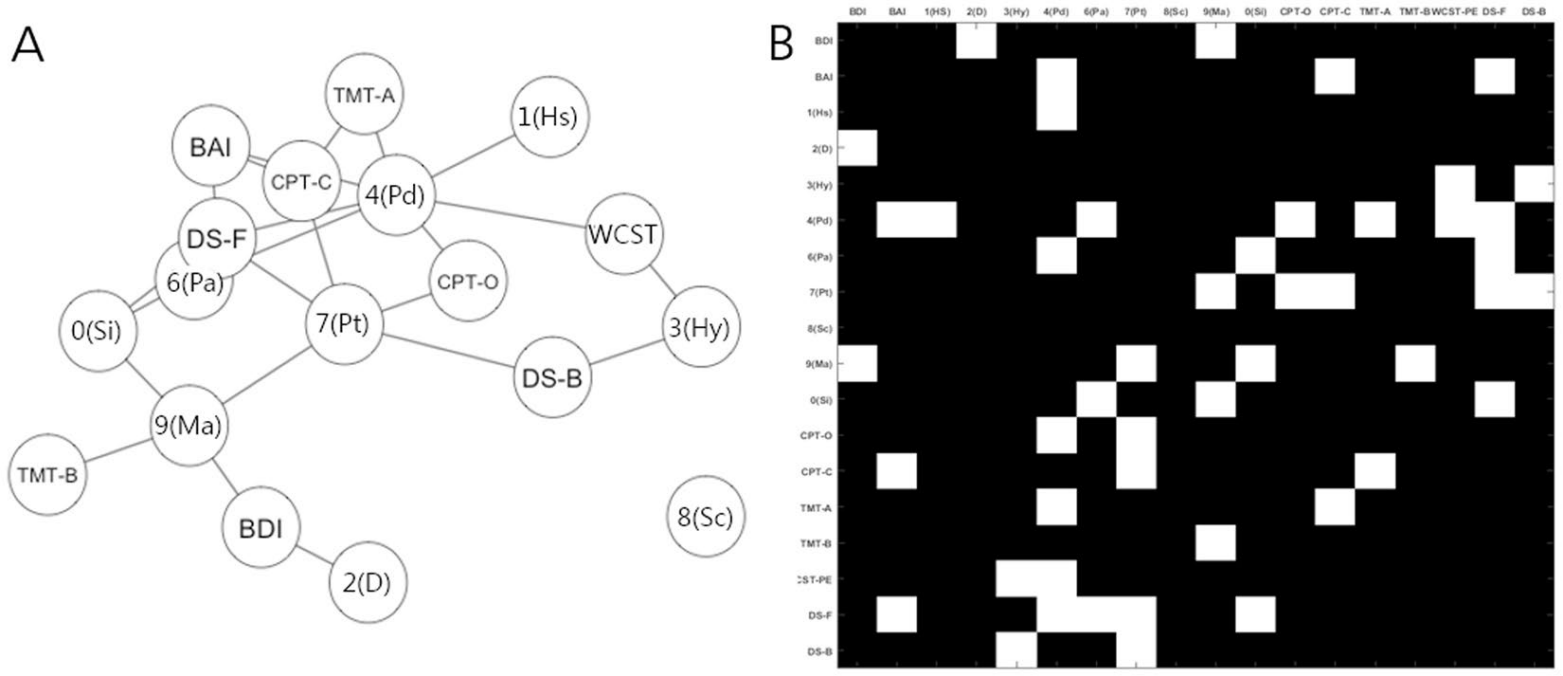
The most influential top 24 edges [top 20% ranked for the ‘edge betweeness centrality’ value, calculated from the undirected-weighted version of intra-individual variability network (K = 0.20–0.21), in more than 45% of participants (*n* = 90)] that connect the 18 nodes of the intra-individual variability network. Visualization was conducted using (**A**) Matlab software and (**B**) R package named *qgraph*. Abbreviations: BAI, Beck Anxiety Inventory total score; BDI, Beck Depression Inventory total score; 1(Hs), MMPI-2 clinical scale 1(Hypochondriasis); 2(D), MMPI-2 clinical scale 2(Depression); 3(Hy), MMPI-2 clinical scale 3(Hysteria); 4(Pa), MMPI-2 clinical scale 4(Psychopathic deviate); 6(Pa), MMPI-2 clinical scale 6(Paranoia); 7(Pt), MMPI-2 clinical scale 7(Psychasthenia); 8(Sc), MMPI-2 clinical scale 8(Schizophrenia); 9(Ma), MMPI-2 clinical scale 9(Hypomania); 0(Si), MMPI-2 clinical scale 0(Social introversion); CPT-O, omission error of the continuous performance test; CPT-C, commission error of the continuous performance test; TMT-A, reaction time of the trail making test part A; TMT-B, reaction time of the trail making test part B; WCST-PE, perseverative error of the Wisconsin card sorting test; DS-F, forward span of the digit span test; DS-B, backward span of the digit span test.

The most influential top 24 edges [top 20% ranked edges across *K* = 0.20–0.21 in more than 45% of participants] are shown in Fig. 4(A) [drawn using the ‘R’ package *qgraph* ver. 1.4.3. (http://sachaepskamp.com/qgraph)] and Fig. 4(B) [plotted using Matlab R2014b]. Except for MMPI-2: 8(Sc) [connected with 0 influential edges], 1(Hs) [having one influential edge connected to MMPI-2: 4(Pd)], 2(D) [connected only to BDI total score] and Trail Making Test part B (TMT-B) reaction time [share one influential edge with MMPI-2: 9(Ma)], most of the clinical-neurocognitive variables shared two or more influential edges with other variables. Of note, node MMPI-2: 4(Pd) had seven influential edges, including BAI total score, MMPI-2: 1(Hs) and 6(Pa), omission error during Continuous Performance Test (CPT), reaction time of Trail Making Test part A (TMT-A), perseverative error of WCST, and forward span score of digit span test. Next, node MMPI-2: 7(Pt) was connected with a total of five other clinical-neurocognitive variables including MMPI-2: 9(Ma), commission error and omission error of CPT, in addition to forward and backward span of the digit span test. Another node, named forward span of digit span test, shared five influential edges only with MMPI-2 clinical scales [4(Pd), 6(Pa), 7(Pt), and 0(Si)] and BAI total score.

**Figure 5:**
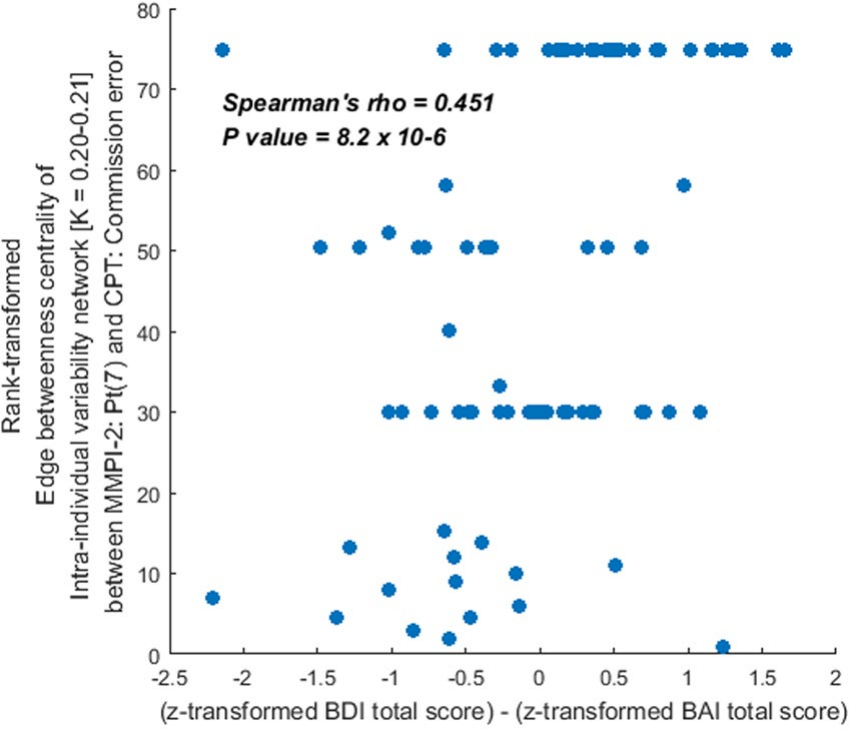
Relationship between the difference of z-transformed total scores for Beck depression inventory (BDI) and Beck anxiety inventory (BAI) [x-axis] and rank-transformed edge betweenness centrality of intra-individual variability network [undirected, weighted version (K = 0.20–0.21)] for the Minnesota multiphasic personality inventory clinical scale (MMPI-2): 7(Pt) and continuous performance test (CPT): commission error [y-axis] (*p* < 0.002 = 0.05/24 for Spearman’s rho [top 24 edges illustrated in Fig. 4.]).

Furthermore, among the 24 influential edges [reflecting the covariance between two different variables], there were eight influential edges mediating nodes [MMPI-2: 4(Pd) and 7(Pt)] that grasped more than four influential edges into another domain [connector between neurocognitive and clinical nodes]. Specifically, an edge reflecting the rank-transformed edge betweenness centrality of covariance between the MMPI-2: 7(Pt) and commission error of CPT demonstrated statistically significant correlation with difference between the ‘z-score transformed BDI total score’ and ‘z-score transformed BAI total score’ (S*pearman’s rho* = 0.451, *p* = 8.2 × 10^–6^; Fig. 5).

## Discussion

To the best of the authors’ knowledge, this is the first reported study to explore the intra-individual covariance-based hierarchical relationship among the clinical [BDI, BAI, MMPI-2 clinical scale]-neurocognitive [commission and omission score of CPT, reaction time for TMT-A and –B, perseverative error of WCST, forward and backward span of digit span test] variables in a campus-based college population. In the sparsity range of K = 0.20–0.21, weighted and undirected version of intra-individual covariance network of clinical-neurocognitive variables satisfied small-worldness and modular organization. Furthermore, using the regional network characteristics of betweenness centrality [=importance of specific node or edge as a mediator comprising the shortcut pathway among various nodes], this study uncovered a reduced influence of strategic planning-cognitive flexibility [=WCST perseverative error] on other clinical-neurocognitive characteristics in more depressive individuals; reports of higher anxiety levels were related to increased control of sociality trait [=MMPI-2 0(Si)] in the intra-individual covariance network. Furthermore, tight coupling of obsession-compulsivity [=MMPI-2 7(Pt)] with impulsivity [=CPT commission error] to mediate the covariance between other clinical-neurocognitive variables as a shortcut edge could predict reports of more anxiety symptoms than complaints of depressive mood.

### Strategic planning (WCST perseverative error) against depressive mood

In this study, participants highly-ranked for the betweenness centrality of WCST perseverative error reported relatively mild depressive mood in terms of BDI total score (Fig. 2(A)). Appropriate strategic planning and flexible cognitive adaptation of hypotheses about current situation in response to the changing feedback would be crucial for the preservation of self-efficacy and timely performance in academic/occupational tasks^33–35^. Conversely, previous suffering or current experience of depressive symptoms has been found to coexist with executive functioning impairment^36–38^. When clinical-neurocognitive characteristics are primarily governed by strategic planning and cognitive flexibility [highly-ranked betweenness centrality of WCST perseverative error], stress resilience can be strengthened and vulnerability to psychopathology reduced^39–41^. With few therapeutic regimens proven to be effective for ameliorating depression-related executive dysfunction^42,43^, clinicians may inform depressive individuals about the enduring nature of cognitive vulnerability for depression while also referring to the therapeutic potential of cognitive training^44^ or theta-burst transcranial magnetic stimulation^45^, among other treatments.

### Influential sociality (MMPI-2 clinical scale Si(0)) and higher anxiety

This study demonstrated elevated betweenness centrality value (=higher rank-transformed centrality) of MMPI-2 clinical scale 0(Si) for participants with higher anxiety (Fig. 2(B)). This result is in line with the Heeren and McNally (2016), in which social introversion-related behavioural tendency such as avoidance and fear of social situations were highly ranked for centrality measurement in the inter-individual psychopathology network for social anxiety disorder. Social interaction style, as reflected in tasks such as following of others’ eye gaze direction^46^ or recognition of facial emotion^47,48^, greatly affects an individual’s susceptibility to anxiety^49^. Moreover, when sociality factor measured using MMPI-2 0(Si) prevails in the hierarchical covariance network of clinical-neurocognitive features, this state might be likened to the trait of sensory processing sensitivity^50^ specifically biased towards nonverbal social cues^51^. Given the consistency of sociality trait within individuals across time, psychoeducation for enduring trait anxiety combined with mindfulness-based stress reduction training could be helpful in reducing and controlling anxiety symptoms^52,53^.

### Tight compulsivity-impulsivity covariance as a shortcut to higher anxiety

In this study, participants who reported more severe levels of anxiety beyond the intensity of depressive mood, revealed highly ranked edge betweenness centrality for covariance between MMPI-2 clinical scale 7(Pt) and CPT commission error (Fig. 4). Although poor impulse control might be shown in a sub-population with a genetic loading for depression by mediation of the serotonin transporter^54^, depressive mood *per se* did not affect CPT performance^55^. Concurrent escalation of compulsivity and impulsivity have been related to substance use disorder in a college population^56^ and suggests the highest clinical severity in patients with obsessive-compulsive disorder^57^. Importance of the compulsivity-impulsivity interplay, reflected as increased edge-betweenness centrality of ‘MMPI-2: 7(Pt)–CPT: commission error’ edge for participants who reported more severe anxiety beyond the intensity of depressive mood, has been also shown valid for anxiety disorders and attention-deficit/hyperactivity disorder^56^. When compulsivity or impulsivity becomes attenuated with symptom-specific cognitive behavioural therapy or brain circuitry-targeted repetitive transcranial magnetic stimulation^58^, the influence of compulsivity-impulsivity covariance as a shortcut mediator among the various clinical-neurocognitive variables might be reduced.

### Limitations

This study has some limitations. First, participants did not undergo DSM-derived evaluation procedures using SCID-I or SCID-NP. Second, as participants were measured for their psychological characteristics at only one time-point, intra-individual comparison of covariance network characteristics varying with changed severity of depressive mood and/or anxiety were not possible. However, regardless of the psychiatric diagnosis, by deciphering the key driver of hierarchical dynamics among the clinical-neurocognitive variables per individual, this study has provided clinicians with a specific point of leverage for non-pharmacological treatment as an initial therapy.

## Conclusions

Applying the format of an intra-individual covariance network into the hierarchical dynamic of clinical-neurocognitive variables per individual, this study demonstrated critical drivers of depressive mood [attenuated influence of strategic planning, measured using the WSCT perseverative error] or anxiety [domination of social introversion/extroversion reflected in the MMPI-2: 0(Si) in addition to the critical influence of covariance between compulsivity (MMPI-2: 7(Pt)) and impulsivity (CPT commission error) as a shortcut component among various clinical-neurocognitive features] in undergraduate and graduate students not receiving psychotropic medication. Further studies to explore intra-individual changes of hierarchical dynamic among the clinical-neurocognitive variables across multiple time-points would allow examination of the value of these components as effective points of therapeutic intervention.

## Methods

### Participants and measures

In total, 90 undergraduate or graduate students (57 females; aged 24.1 ± 4.3 (mean ± SD)) who were not prescribed any psychotropic medication at the time of study enrolment volunteered for this study. Subjective recent experiences of depressive mood and anxiety were measured using the total score of BDI^59^ and total score of BAI^60^, respectively. More diverse psychopathology and sociality were systematically assessed using the MMPI-2 [*T-scores* for nine clinical scales: 1(Hs; hypochondriasis), 2(D; depression), 3(Hy; hysteria), 4(Pd; psychopathic deviate), 6(Pa; paranoia), 7(Pt; psychasthenia), 8(Sc; schizophrenia), 9(Ma; hypomania), and 0(Si; social introversion) used in this study]^61,62^.

Moreover, diverse domains of nonverbal neurocognitive performance were assessed with the omission error (the number of failures in responding to a target stimulus; related to inattentiveness) and commission error(responses to a non-target stimulus; measuring impulsivity) of CPT^63^, the reaction times of TMT-A (psychomotor speed) and TMT-B (visuomotor sequencing and set shifting)]^64^, the perseverative error of WCST (the number of erroneous sorting events that occurred when participant used the same principle applied during their previous sorts; concept formation, set-shifting and strategic planning)^65–67^ and digit span from the Korean version of the Wechsler Adult Intelligence Scale [DS; forward span (sustained attention) and backward span (working memory)]^68^. The Institutional Review Board at Seoul National University Hospital approved the current study. Written informed consent was obtained from all subjects after the procedures had been fully explained, and all methods were performed in accordance with the relevant guidelines and regulations.

### Construction of the intra-individual covariance network

We adapted a total of 18 clinical-neurocognitive variables into an intra-individual covariance network. First, group-wise (*n* = 90) mean and standard deviation per variable was calculated^27,28^. To ensure that positive z-scores indicated a better condition, all of the z-values other than forward or backward scores of the digit span test were multiplied by -1. Second, all scores per participant were z-score transformed using the group-wise mean and standard deviation. Third, the difference (=covariance) between two z-transformed variables was converted into an edge of intra-individual covariance network of clinical-neurocognitive features using the formula ‘${{\rm{edge}}}_{\mathrm{covariance}(i,j)}=1/({e}^{{(z(i,t)-z(j,t))}^{2}})$’, in which *z(i, t)* and *z(j, t)* denote z-transformed values of specific clinical-neurocognitive variables for participant *t*, to finally construct a total of 90 intra-individual covariance networks of clinical-neurocognitive features^30,31^.

### Network analyses: Global network characteristics

Global-regional characteristics of the intra-individual covariance networks of clinical-neurocognitive features (refer to the previous section) were explored with graph theory approach using the Brain Connectivity Toolbox (https://www.nitrc.org/projects/bct/) in Matlab R2014b (https://kr.mathworks.com). First, each network matrix was trimmed to survive only top 10–30% ranked edges for edge strength [using ‘threshold_proportional.m^32^’]. Second, to examine the presence of and to clearly identify the range of sparsity [*K*; the percentage of edge presence compared to the possible number of edges in a network], in which small-worldness and modular organization of given intra-individual covariance networks satisfied, four global network characteristics were calculated as below: (a) normalized clustering coefficient [*gamma*; was measured using ‘clustering_coef_wu.m^69^, per node and were averaged over a total of 18 nodes, and finally were normalized using the same variable calculated from the 10,000 degree-constrained random networks produced from the original network with ‘randmio_und.m^70^’], (b) normalized characteristic path length [lambda; after transforming the original network into the shortest path-length between nodes using ‘distance_wei.m^32^’, the characteristic path length among nodes in a given network were calculated using ‘charpath.m^32^’, and finally were normalized using the same variable calculated from the 10,000 random network alike in case of (a)], (C) small-worldness [*sigma* = *gamma*/*lambda*]^32^, and (d) modularity [*Q*; a statistic that quantifies the degree to which the network may be subdivided into more clearly delineated communities, averaged over 500 runs of trial using ‘modularity_und.m^71,72^’ considering the heuristic nature of the algorithm used].

As a result, in the sparsity range of *K* = 0.20–0.21, more than 95% of intra-individual covariance networks satisfied the (1) small-world organization (*sigm*a > 1), (2) modular organization (Q > 0.3; *K* = 0.10–0.21), and showed (3) network connectedness [more than 80% ( > 15) of nodes from a total of 18 nodes were connected to at least one other node; *K* = 0.20–0.21]^73^. Therefore, this sparsity range of *K* = 0.20–0.21 became the focus of the next exploration for regional network characteristics.

### Network analyses: Regional network characteristics

Using the connection-length matrix, an inverse of original intra-individual covariance network matrix (*K* = 0.20–0.21), ‘betweenness centrality [indicates the frequency at which a node is located in the path of the shortcut that connects two different nodes in a network; calculated using ‘betweenness_wei.m’]^74^’ per node was calculated. In a scale-free network, distribution of the centrality value does not follow normal distribution. Thus, prior to correlation analyses with clinical characteristics (BDI total score and BAI total score), these betweenness centrality values were rank-transformed per participant using the ‘tiedrank.m’ function of Matlab R2014b.

Additionally, ‘edge betweenness centrality [the fraction of a given edge being a component of shortest paths connecting different nodes in a network; measured using ‘edge_betweenness_wei.m’]^74^, values were also gathered from the connection-length version of the original intra-individual covariance network matrix. After having been rank-transformed and being averaged over the sparsity range of *K* = 0.20–0.21 per participant, the most influential top 24 edges [top 20% ranked edges in more than 45% of participants] were retrieved^75,76^.

### Statistical analyses

Descriptive statistics of mean and standard deviation values were calculated using the ‘mean.m’ and ‘std2.m’ functions of Matlab R2014b, respectively. To explore the correlation between the rank-transformed betweenness centrality values and clinical symptom severity [BDI total score for depressive mood; BAI total score for anxiety], Spearman’s correlation coefficients were calculated [using the ‘spearman’ option of the ‘corr.m’ function]; considering the exploratory nature of the analysis, the statistical threshold of significance was set at *p* < 0.01. Furthermore, to challenge the role of influential edges as a reflection of clinical symptom dynamics [reported severity of depressive mood versus anxiety] per participant, Spearman’s correlation coefficients between the rank-transformed edge betweenness centrality values [the most influential top 24 edges; top 20% ranked edges in more than 45% of participants] versus difference between ‘z-transformed BDI score’ and ‘z-transformed BAI score’ were assessed (*p* < 0.002 = 0.05/24).

### Data Availability Statement

The authors will make materials, data and associated protocols promptly available to readers without undue qualifications in material transfer agreements.
